# Immunomodulatory Drugs in the Context of Autologous Hematopoietic Stem Cell Transplantation Associate With Reduced Pro-tumor T Cell Subsets in Multiple Myeloma

**DOI:** 10.3389/fimmu.2018.03171

**Published:** 2019-01-21

**Authors:** Giulia Di Lullo, Magda Marcatti, Silvia Heltai, Cristina Tresoldi, Anna Maria Paganoni, Claudio Bordignon, Fabio Ciceri, Maria Pia Protti

**Affiliations:** ^1^Tumor Immunology Unit, Division of Immunology, Transplantation and Infectious Diseases, Istituto di Ricovero e Cura a Carattere Scientifico (IRCCS) San Raffaele Scientific Institute, Milan, Italy; ^2^Hematology and Bone Marrow Transplantation Unit, IRCCS San Raffaele Scientific Institute, Milan, Italy; ^3^Molecular Hematology Unit, IRCCS San Raffaele Scientific Institute, Milan, Italy; ^4^Laboratory for Modeling and Scientific Computing (MOX), Dipartimento di Matematica,Politecnico di Milano, Milan, Italy; ^5^Vita-Salute San Raffaele University, Milan, Italy

**Keywords:** multiple myeloma, immunomodulatory drugs, autologous hematopoietic stem cell transplantation, bone marrow, anti-tumor and pro-tumor T cell subsets

## Abstract

Immunomodulatory drugs (IMiDs) are effective therapeutics for multiple myeloma (MM), where in different clinical settings they exert their function both directly on MM cells and indirectly by modulating immune cell subsets, although with not completely defined mechanisms. Here we studied the role of IMiDs in the context of autologous hematopoietic stem cell transplantation on the T cell subset distribution in the bone marrow of newly diagnosed MM patients. We found that after transplantation pro-tumor Th17-Th1 and Th22 cells and their related cytokines were lower in patients treated with IMiDs during induction chemotherapy compared to untreated patients. Of note, lower levels of IL-17, IL-22, and related IL-6, TNF-α, IL-1β, and IL-23 in the bone marrow sera correlated with treatment with IMiDs and favorable clinical outcome. Collectively, our results suggest a novel anti-inflammatory role for IMiDs in MM.

## Introduction

Multiple Myeloma (MM) is a plasma cell neoplastic disorder primarily localized in the bone marrow (BM), where interactions between neoplastic cells and cells within the tumor microenvironment (i.e., mesenchymal stromal cells, endothelial cells, osteoblasts, osteoclasts, and immune cells) mediate disease development and progression (1–3).

Th cells play fundamental regulatory function in adaptive immune responses and in antitumor immunity being, according to their secretory cytokine profile, either anti-tumor or pro-tumor (4). In MM imbalanced Th cell polarization and subset distribution in the BM niche largely impact on disease progression (5).

Immunomodulatory drugs (IMiDs), namely thalidomide and its derivatives lenalidomide and pomalidomide, are major therapeutics in the treatment of MM (6), where their efficacy has been demonstrated in newly diagnosed patients either eligible or ineligible for autologous hematopoietic stem cell transplantation (ASCT), in the maintenance setting after ASCT and in refractory/relapsed disease (6).

In MM, in addition to anti-proliferative and pro-apoptotic effects on malignant cells, IMiDs exert immune regulatory function and interfere with tumor microenvironment interactions (7). *In vitro* IMiDs enhanced T cell proliferation, IL-2 and IFN-γ secretion and NK cell activation (8, 9), lenalidomide improved immune checkpoint blockade-induced immune responses (10) and inhibited T regulatory cell (Treg) proliferation and suppressor function (11). *In vivo* lenalidomide augmented (i) vaccine responses and endogenous anti-tumor immunity (12), (ii) the number of central and effector memory CD8^+^ T cells, Tregs and CD14^+^CD15^+^ myeloid derived suppressor cells in patients that received lenalidomide as monotherapy or in combination with other treatments (13), (iii) the number of Tregs in patients in consolidation/maintenance therapy (14, 15), (iv) anti-myeloma specific T cell responses in patients that received lenalidomide as consolidation therapy after ASCT (16), and (v) the number of IFN-γ and IL-21 producing cells in the setting of maintenance therapy (17). Treatment with lenalidomide was also associated with impaired long-term thymic reconstitution and decreased number of CD4^+^ and CD8^+^ effector terminally differentiated T cells (14, 15) and reduced PD-1 expression on T cells in the maintenance setting (18).

A prospective randomized trial comparing induction regimens prior to ASCT including or not thalidomide (i.e., bortezomib-thalidomide-dexamethasone) vs. bortezomib-cyclophosphamide-dexamethasone) recently reported significantly higher overall clinical response rate in the bortezomib-thalidomide-dexamethasone arm (19).

In this study, we evaluated in newly diagnosed MM patients the impact of IMiDs used in the induction chemotherapy preceding ASCT on the distribution of T cell subsets and related cytokines in the BM after transplantation and whether changes in those immunological parameters correlated with the clinical outcome.

## Materials and Methods

### Subjects and Samples

Forty-four newly diagnosed MM patients, who had been hospitalized at the Hematology Department of our Institution and received ASCT as first line therapy, were selected for the study. The Institutional Ethics Committee (Comitato Etico Fondazione Centro San Raffaele, Istituto Scientifico Ospedale San Raffaele) had approved the study protocol and written informed consent was obtained from all donors. Clinical data and information on the induction chemotherapy received by each patient are reported in Table 1. BM mononuclear cells were isolated by density gradient centrifugation with Ficoll-Paque^TM^ Plus (GE Healthcare, Uppsala, Sweden), frozen in fetal bovine serum (Lonza, Milan, Italy) + 10% DMSO (Sigma-Aldrich, Milan, Italy), and stored according to standardized operating procedures by the Institutional Biobank. BM mononuclear cells were used after thawing and viable cell counting. BM sera were taken and collected according to standardized operating procedures by the Institutional Biobank. Briefly, non-heparinized BM blood (5–7 ml) was incubated for 1 h at room temperature to achieve complete clotting. Then samples were centrifuged at 1,600 g without brake for 10 min at 4°C and supernatants transferred in cryovials and stored in liquid nitrogen until use.

**Table 1:** Characteristics of the patients.

**Patient ID**	**Sex^a^**	**Age^a^**	**% PC in BM aspirate^a^**	**% PC in BM biopsy^a^**	**Ig type^a^**	**DS^a^**	**ISS^a^**	**Anemia^a^**	**Hyper-calcemia^a^**	**Renal dysfunction^a^**	**Bone lesions^a^**	**Induction chemotherapy (n. of cycles x drug combination)^b^**	**Clinical response at 3 months post-ASCT^c^**
**INDUCTION CHEMOTHERAPY WITHOUT IMiDs**													
PZ10000419	M	51	46	58	IgGk	IIA	I	+	–	–	–	3 x VD	VGPR
PZ10000456	M	40	62	n.a.	IgGk	IIIA	II	+	+	–	+	4 x VCD	VGPR
PZ10005342	M	65	n.a.	12	IgGk+IgAk	IIIA	II	–	–	–	+	6 x VCD	PR
PZ11000002	F	57	35	42	IgGk	IIIA	I	+	–	–	+	4 x VCD	VGPR
PZ11000005	M	53	52	46	IgGk	IIIA	I	–	–	–	+	4 x VCD	VGPR
PZ11000126	M	63	72	55	IgGk+k	IIIA	II	+	–	–	+	4 x VCD	PR
PZ11000194	F	64	85	n.a.	IgGλ	IIA	I	+	–	–	–	4 x VCD	VGPR
PZ12000148	M	58	81	62	IgGk	IIIA	III	+	–	+	+	4 x VD	PR
PZ12000149	F	64	62	70	IgGk	IIIB	III	+	–	+	+	3 x VD	CR
PZ14000034	F	69	n.a.	n.a.	IgGk	n.a.	n.a.	n.a.	n.a.	n.a.	+	3 x VAD	PR
**INDUCTION CHEMOTHERAPY WITH IMiDs**													
PZ10000388	M	50	40	53	IgGk	IIA	I	+	–	–	–	4 x Ln/Dx	REF
PZ10000451	F	58	26	46	IgAk	IIIA	I	+	–	–	+	4 x Ln/Dx, 4 x VCD	PR
PZ11003115	M	40	78	95	IgGλ	IIIA	III	+	–	+	+	3 x VTD	VGPR
PZ11005967	M	36	14	28	IgGk	IA	I	–	–	+	–	3 x VTD	PR
PZ11005971	M	65	n.a.	42	IgGk	IIIA	I	+	–	–	+	3 x VTD	CR
PZ11005996	M	57	93	65	k	IB	III	+	+	+	–	3 x VTD	CR
PZ12000003	F	55	85	85	IgAλ	IIIA	II	+	–	–	+	4 x VTD	CR
PZ12000058	M	34	n.a.	50	IgGk	IIA	I	–	–	–	–	3 x VTD	CR
PZ12000123	F	70	40	60	IgGλ	IIA	II	–	–	–	+	2 x Ln, 2 x PAD	VGPR
PZ13000015	F	61	68	52	IgAλ	IIIA	II	–	–	–	–	1 x VTD	CR
PZ13000024	M	62	18	24	k	IIIA	I	–	–	–	+	3 x VTD	CR
PZ13000044	M	60	25	n.a.	IgGλ	IIA	I	+	–	–	+	4 x VTD	PR
PZ13000066	F	50	60	48	IgGλ	IIIA	II	+	–	–	+	5 x VTD	CR
PZ13000070	F	52	n.a.	7	IgGk	IIIA	I	+	–	–	+	3 x VTD	CR
PZ13000079	M	68	19	n.a.	IgGk	IIA	II	–	–	–	–	3 x VTD	PR
PZ13000102	M	47	26	n.a.	IgGλ	IIA	I	–	–	–	n.a.	3 x VTD	REF
PZ13000168	M	40	54	n.a.	k	IIIA	II	–	–	+	+	2 x VTD	VGPR
PZ13000144	M	56	7	55	IgGk	IIIA	I	+	–	–	+	2 x VTD	VGPR
PZ14000009	F	58	45	n.a.	k	IIIB	III	+	+	+	+	4 x VTD	CR
PZ14000039	F	65	n.a.	50	IgGk	IIIA	I	–	–	–	+	3 x VTD	VGPR
PZ14000051	F	60	36	32	IgGk	IA	II	–	–	–	–	4 x VTD	VGPR
PZ14000060	M	50	17	26	IgGλ	IIIA	I	–	–	–	–	4 x VTD	VGPR
PZ14000102	F	65	n.a.	90	IgGk	IIIA	III				+	3 x VTD, 8 x Ln/Dx, 4 x VAD	VGPR
PZ14000106	M	61	7	48	IgGk	IA	I	–	–	–	–	1 x VTD	CR
PZ14000125	M	54	n.a.	>90	IgAλ	IIIA	II	–	–	–	n.a.	3 x VTD	VGPR
PZ14000157	M	63	64	42	IgGk	IIIA	II	+	–	–	+	4 x VTD	VGPR
PZ14000172	F	61	n.a.	70	λ	IA	II	+	–	+	+	3 x VTD	CR
PZ160019	M	40	n.a.	60	IgAk	IIIA	II	+	–	–	+	4 x VTD	CR
PZ170070	F	72	28	42	λ	IB	II	+	–	+	–	5xV(T)D	CR
PZ160112	M	65	32	68	IgGk	IIIA	II	+	–	+	+	4 x VTD	PR
PZ14000138	F	62	31	n.a.	IgGk	IIIA	I	–	–	–	+	5 x VTD	VGPR
PZ15000120	F	67	37	60	IgGλ	IIA	II	+	–	–	+	6 x VTD	VGPR
PZ15000136	F	68	14	23	IgGk	IA	I	–	–	–	+	2 x VTD	CR
PZ160062	M	50	n.a.	90	IgAk	IIIA	III	+	–	–	+	4 x VTD	CR

a *Clinical data at the time of MM diagnosis*.

b *Induction chemotherapy preceding the first ASCT received by the patient*.

c *Clinical response was evaluated following the International Myeloma Working Group consensus criteria for response and minimal residual disease assessment in multiple myeloma (20)*.

*DS, Durie-Salmon Staging System; F, female; ISS, International Staging System; Ln, lenalidomide; Ln/Dx, lenalidomide/dexamethasone; M, male; n.a., not available; PAD, bortezomib (P) doxorubicin (A) dexamethasone (D); PC, plasma cells; VD, bortezomib (V) dexamethasone (D); VAD, vincristine (V) doxorubicin (A) dexamethasone (D); VCD, bortezomib (V) cyclophosphamide (C) dexamethasone (D); VTD, bortezomib (V) thalidomide (T) dexamethasone (D); CR, complete response; PR, partial response; REF, refractory disease; VGPR, very good partial response*.

***Dosages and schedule:** VCD (21) (28-day cycle): Bortezomib, 1.3 mg/m^2^ (days 1, 8, 15, 22); Dexamethasone, 40 mg (days 1-2, 8-9, 15-16, 22-23); Cyclophosphamide, 500 mg/m^2^ (days 1, 8, 15)*.

*VTD (22) (21-day cycle): Bortezomib, 1.3 mg/m^2^ (days 1, 4, 8, 11); Dexamethasone, 40 mg (days 1-4, 8-11), Thalidomide, 50 mg for the first 14 days and then 100 mg (days 1-21). Thereafter, starting from the second cycle 200 mg*.

*VD (23) (21-day cycle): Bortezomib, 1.3 mg/m^2^ (days 1, 4, 8, 11); Dexamethasone, 40 mg (days 1–4, 8–11)*.

*Len/Dex (24) (28-day cycle): Lenalidomide, 25 mg/die (days 1-21/28); Dexamethasone, 40 mg (days 1, 8, 15, 21)*.

### Flow Cytometry Analysis

The following antibodies were used: Pacific blue-conjugated CD3 (clone UCHT1) from Dako Cytomation (Cernusco sul Naviglio (MI), Italy); PerCP-conjugated CD4 (clone L200), PE-conjugated CD25 (clone 2A3), PE-conjugated IL-13 (clone JES10-5A2), and APC-conjugated IL-5 (clone TRFK5), all from BD Biosciences (Milan, Italy); PE-Cy7-conjugated CD127 (clone R34.34) from Beckman Coulter (Cassina De' Pecchi (MI), Italy); PE-Cy7-coniugated IL-22 (clone 22URTI) from eBioscience (Milan, Italy); FITC-conjugated CD3 (clone UCHT1), FITC-conjugated IFN-γ (clone B27), Alexa Fluor488-conjugated IL-4 (clone MPA-25D2), Alexa Fluor647-conjugated IL-17 (clone BL168), all from Biolegend (Milan, Italy). For the study of the percentage of T cells secreting distinct effector cytokines, thawed BM mononuclear cells were rested at room temperature for 30 min before viable cell counting and then cultured at a cell density of 2 × 10^6^ cells/ml for 5 h in x-vivo-15 medium (Lonza), supplemented with penicillin (100 U/ml), streptomycin (100 U/ml), and 3% human serum type AB (Lonza), in the absence or the presence of 50 ng/ml PMA (phorbol-12-myristate-13-acetate) and 1 μg/ml ionomycin (both from Sigma-Aldrich) to induce cytokine production. After 2 h, 10 μg/ml brefeldin A (Sigma-Aldrich) was added to both unstimulated and stimulated cells. After stimulation, cells were harvested, washed and stained first for the surface markers CD3 and CD4 and then for intracellular cytokines, using a kit from BD Biosciences, following the manufacturer's instructions. In detail, for intracellular cytokine staining cells were fixed, permeabilized with Cytofix/Cytoperm buffer, and incubated with antibodies diluted in Perm/Wash buffer. Percentages of cells positive for intracellular cytokine expression were referred to total CD3^+^ T cells because a clear-cut distinction between CD4^+^ and CD4^−^ T cells was hampered by the downregulation of surface CD4 expression on T cells stimulated with PMA + ionomycin (Figure S1). For Treg staining, cells were first stained for the surface antigens CD3, CD4, CD25, and CD127 and then fixed, permeabilized, and stained for the nuclear transcription factor FoxP3 with the dedicated FoxP3/Transcription Factor Staining Buffer from eBioscience, according to the manufacturer's instructions. Percentages of Treg cells, identified as CD3^+^CD4^+^CD127^−^CD25^+^FoxP3^+^ cells, were also referred to total CD3^+^ T cells. Cells were analyzed using a FACSCanto flow cytometer (BD Biosciences) and data were illustrated by FlowJo software (Tree Star Inc., Ashland, OR, USA).

### Cytokine Measurements

Cytokines in BM sera were measured using ELISA kits from Mabtech (Milan, Italy) (i.e., IL-1β, TNF-α, IFN-γ, IL-4, IL-5, IL-6, IL-13, IL-17, IL-22, and IL-23), following the manufacturer's instructions.

### Statistical Analysis

Statistical significance was determined with Mann Whitney U test and Wilcoxon Signed-Rank Test, as reported in the figure legends. In addition, to evaluate the equality of variances for the values in the IMiD-treated vs. the IMiD-untreated group of patients we performed the Levene's test with no statistical evidence to support difference between the variance of the distributions. Statistical analyses were performed with an alpha level of 5% using GraphPad Prism version 5.0 for Mac (GraphPad Software), thus values of *p* < 0.05 were considered significant.

## Results

We studied forty-four newly diagnosed MM patients, who had received ASCT as frontline therapy and whose clinical features are detailed in Table 1. Patients were administered an induction chemotherapy, most often consisting of a bortezomib-based poly-chemotherapy (including or not thalidomide or, less often, lenalidomide) followed by stem cell mobilization with cyclophosphamide and G-CSF, high-dose melphalan and a single or tandem ASCT (see Table 1 for dosage and schedule details).

**Figure 1:**
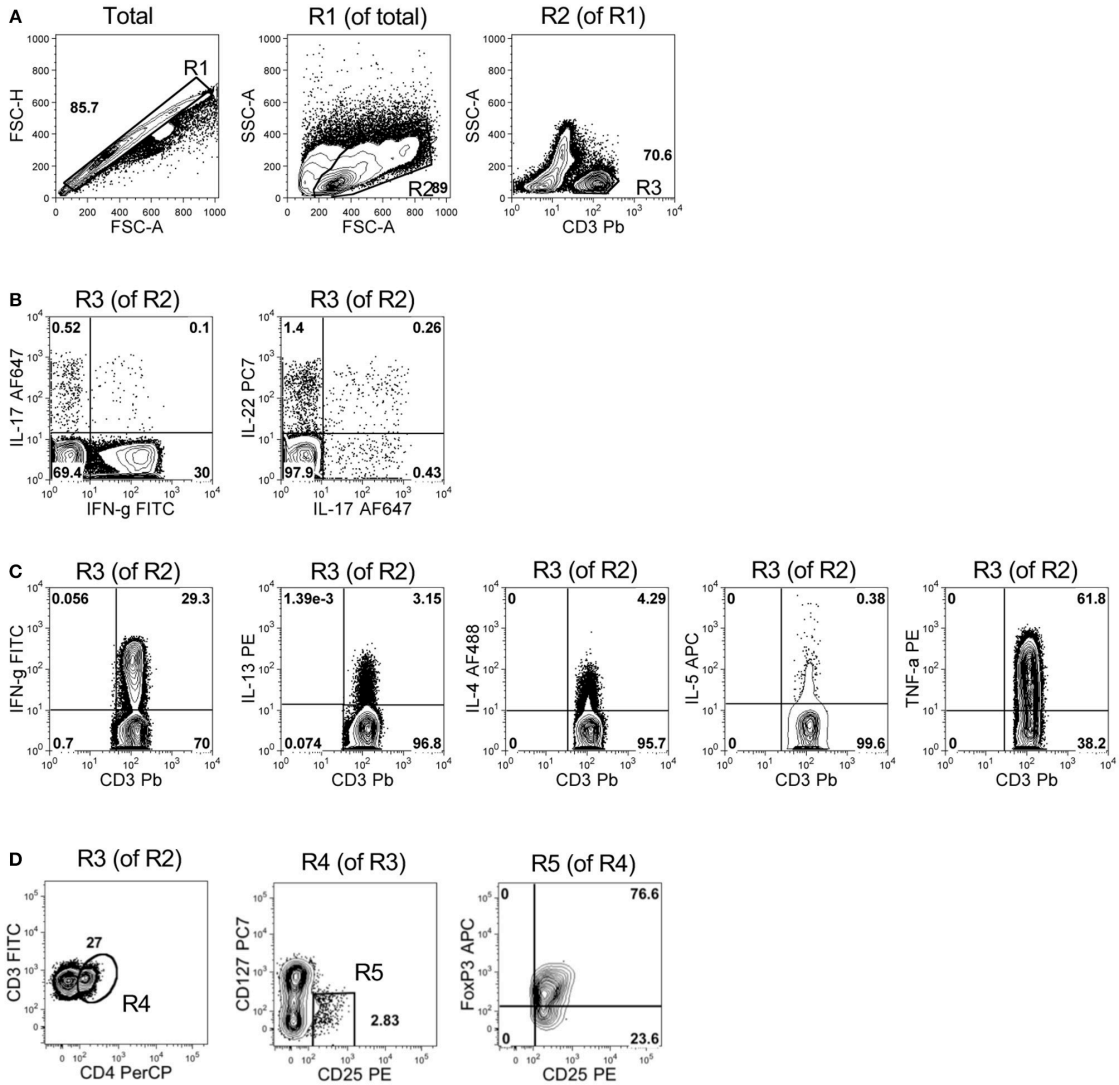
Gating strategy for immunophenotypic analyses in representative samples of BM mononuclear cells. **(A)** Panels represent, from left to right: the pulse geometry R1 gate (in FSC-A x FSC-H dot plot of all the analyzed cells) used to exclude doublets, the morphology-based R2 gate of leukocytes (in FSC-A x SSC-A dot plot of R1-gated cells) and the R3 gate of T cells (i.e., CD3^+^ cells of R2-gated leukocytes). **(B)** Left panel: quadrant gates defining the expression and the percentage of IL-17 and IFN-γ in R3-gated CD3^+^ T cells; right panel: quadrant gates defining the expression of IL-17 and IL-22 in R3-gated CD3^+^ T cells. **(C)** Panels represent, from left to right, the quadrant gates defining expression and the percentage of IFN-γ^+^, IL-13^+^, IL-4^+^, IL-5^+^, and TNF-α^+^ cells in R3-gated CD3^+^ T cells. **(D)** Dot plots showing the gates used to identify and enumerate the percentage of Treg cells (CD4^+^CD127^−^CD25^+^FoxP3^+^) in R3-gated CD3^+^ T cells. From left to right: R4 gate represents the percentage of CD4^+^ cells among total CD3^+^ T cells (i.e., R3-gated cells); R5 gate represents the percentage of CD127^−^ cells among total CD4^+^ T cells (i.e., R4-gated cells); the upper right quadrant gate in the CD25 x FoxP3 dot plot identifies *bona fide* Treg cells (i.e., CD25^+^FoxP3^+^ T cells in R5-gated CD4^+^CD127^−^ T cells). Numbers within the plots represent percentages of R gates **(A,D)** and quadrant statistics **(B–D)**.

BM mononuclear cells were analyzed by flow cytometry for intracellular cytokine staining of the T cell subset-distinctive cytokine patterns or directly for Treg specific markers within the T cell fraction (see Figure 1 for gating strategy).

**Figure 2:**
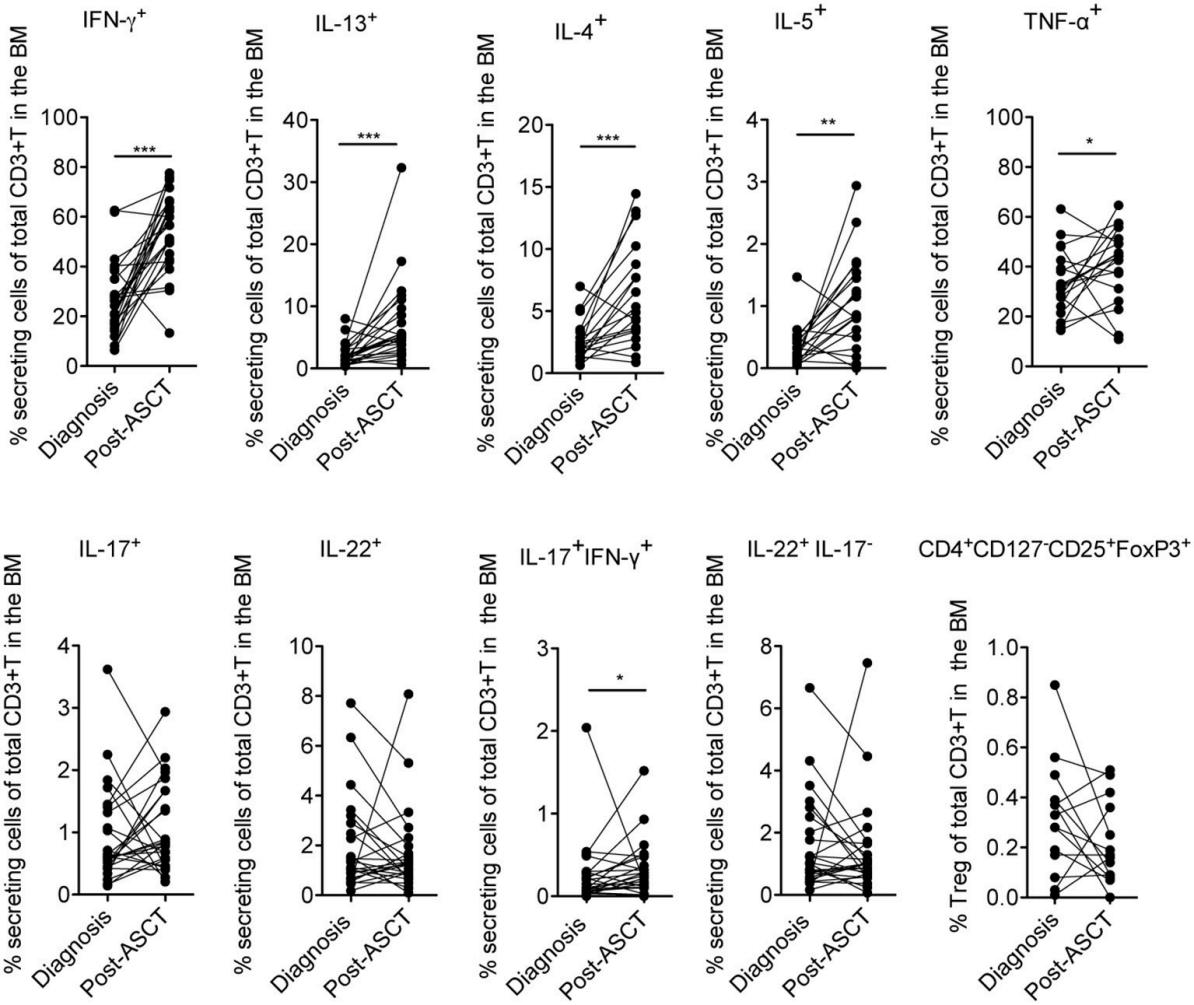
Frequency of distinctive cytokine secreting T cells and Tregs in newly diagnosed MM patients measured in paired BM samples at diagnosis and at 3 months after ASCT. The frequency of CD3^+^ T cells secreting IFN-γ, IL-13, IL-17, IL-22 (*n* = 25), IL-4, and IL-5 (*n* = 19), TNF-α (*n* = 18) and CD3^+^ T cells expressing a Treg phenotype (i.e., CD4^+^CD127^−^CD25^+^FoxP3^+^) (*n* = 14) are reported. Responses significantly different by Wilcoxon Signed-Rank Test are indicated as: ^*^p < 0.05, ^**^0.001 < *p* < 0.01 and ^***^p < 0.001.

**Figure 3:**
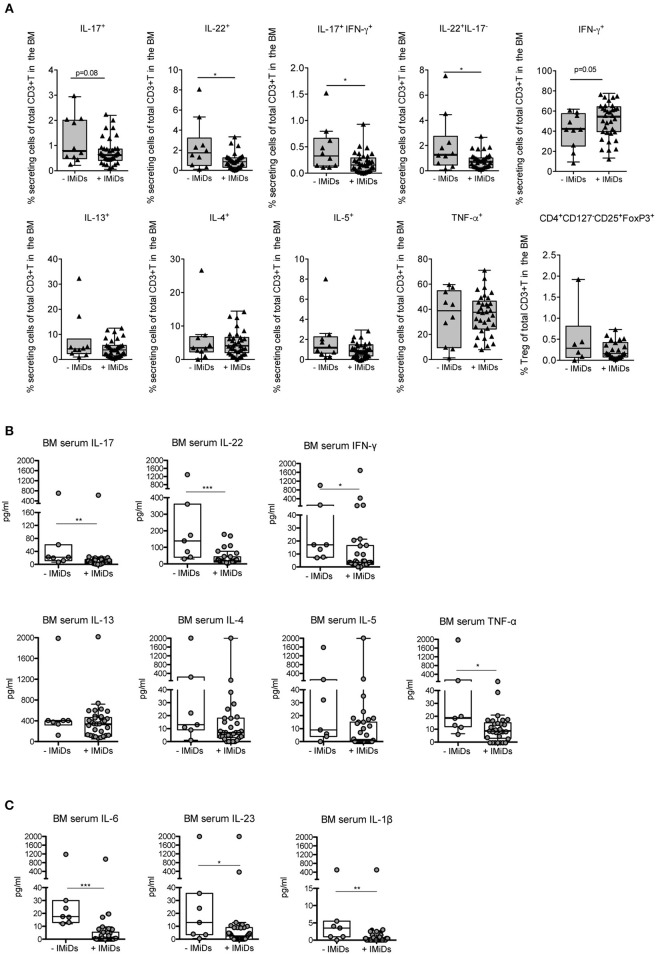
Effects of IMiD treatment on *ex-vivo* distribution of T cell subsets and related cytokines in the BM of MM patients at 3 months after ASCT. **(A)** Percentages of CD3^+^ T cells secreting the indicated cytokines and CD3^+^ T cells expressing a Treg phenotype (i.e., CD4^+^CD127^−^CD25^+^FoxP3^+^), as assessed by surface and intracellular staining analyses, in the BM of patients grouped based on the absence (–IMiDs, *n* = 10 for T cells and *n* = 6 for Tregs) or the presence (+IMiDs, *n* = 34 for T cells and *n* = 23 for Tregs) of IMiDs in the induction chemotherapy. Data from each patient are represented as black-filled triangles. **(B,C)** BM serum levels of the indicated cytokines in MM patients grouped as above (-IMiDs, *n* = 7; +IMiDs, *n* = 31). Data from each patient are represented as gray-filled circles. Responses significantly different by Mann-Whitney test are indicated as: ^*^p < 0.05, ^**^0.001 < *p* < 0.01 and ^***^p < 0.001.

Firstly, we measured the frequency of distinct effector cytokine-secreting T and Treg cell subsets in paired samples at diagnosis and 3 months after ASCT (i.e., a time point at which immune cells are mostly reconstituted), to evaluate potential changes of the T cell subset distribution occurring early after transplantation as a consequence of the complete therapy received by the patients (i.e., induction regimen including or not IMiDs, stem cell mobilization, chemotherapy and ASCT). We found that T cells secreting Th1 (i.e., IFN-γ and TNF-α) and Th2 (i.e., IL-13, IL-4, and IL-5) cytokines were significantly increased after ASCT compared to those at diagnosis as well as T cells coproducing IL-17 and IFN-γ, whereas T cells secreting IL-17, IL-22, and Tregs (CD4^+^CD127^−^CD25^+^FoxP3^+^) were not (Figure 2). Secondly, to evaluate the impact of IMiDs used in the induction regimen, we focused on the frequency of the T cell subsets in the post-ASCT time point and performed analyses in patients grouped based on the absence or the presence of IMiDs in the induction chemotherapy. We found that IL-17^+^IFN-γ^+^ (i.e., mostly Th17-Th1, see Figure S1) and IL-22^+^IL-17^−^ (i.e., mostly Th22, see Figure S1) T cells were significantly lower in the IMiD-treated compared to untreated patients (Figure 3A). Remarkably, increased frequency of Th17-Th1 and Th22 cell subsets correlated with development of symptomatic MM and worse prognosis (25, 26), respectively. Indeed, poly-functional Th17-Th1 were found increased in the BM of MM patients compared to those of patients with pre-neoplastic gammopathy (25), and we previously reported increased frequency of Th22 cells in the blood and BM of patients with stage III disease at diagnosis and refractory/relapsed disease compared to those of asymptomatic patients or patients with stage I/II disease (26). Total IL-17^+^ and IL-22^+^ T cells were also lower within BM T cells from IMiD-treated compared to untreated patients (Figure 3A). In agreement with previous reports (9, 13), IFN-γ^+^ secreting cells were higher in the IMiD-treated compared to the untreated group (Figure 3A), whereas T cells secreting the other cytokines (i.e., IL-13, IL-4, IL-5, and TNF-α) and Tregs were comparable between the two groups (Figure 3A).

We then measured the levels of the same cytokines in the BM sera. We found that, as a result of the complete therapy received by the patients, in paired samples at diagnosis and at 3 months after ASCT IFN-γ, IL-13, IL-4, IL-5, TNF-α, IL-17, and IL-22 were all significantly increased at 3 months after transplantation (Figure S2A). In agreement with the T cell data, when we considered the levels of the cytokines in the BM sera after ASCT in patients grouped based on the absence or the presence of IMiDs in the induction chemotherapy, we found that the levels of IL-17 and IL-22 were significantly lower in the BM sera of IMiD-treated vs. untreated patients, and those of Th2 cytokines (i.e., IL-13, IL-4 and IL-5) were not (Figure 3B). On the contrary, the levels of IFN-γ and TNF-α were both significantly lower in IMiD-treated patients (Figure 3B), suggesting that the total amount of these cytokines in the BM sera after transplantation possibly depends on immune cells, other than T cells, which may be differentially targeted by the drugs used in the two induction chemotherapy regimens.

Next, we measured the BM serum levels of cytokines implicated in Th17-Th1 and Th22 cell polarization/expansion, namely, in addition to TNF-α, IL-6, IL-1β, and IL-23 (27). We did not find any significant change in the level of these cytokines when comparing paired BM sera at diagnosis and at 3 months after ASCT (Figure S2B). Interestingly, these cytokines were all significantly reduced in IMiD-treated vs. untreated patients in the BM sera after ASCT (Figure 3C).

Together, these data show that IMiD-including therapies are associated with a lower frequency in the BM at 3 months after transplantation of pro-tumor Th17-Th1 and Th22 cells and of the serum levels of their distinctive cytokines and cytokines implicated in their polarization/expansion.

**Figure 4:**
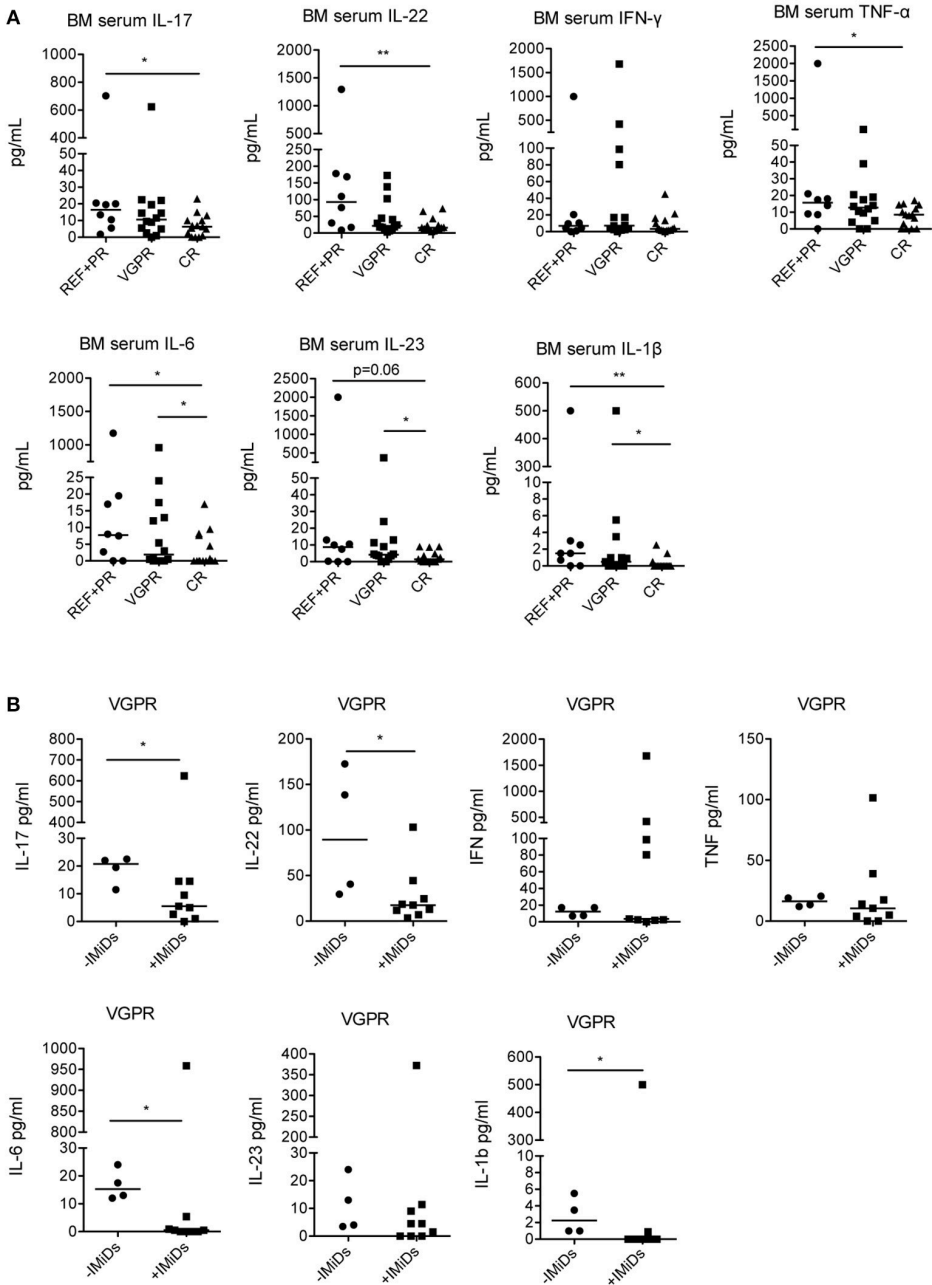
Correlation between cytokine levels in the BM at 3 months after ASCT, clinical outcome and induction chemotherapy regimens. **(A)** MM patients (*n* = 37) were grouped based on the clinical status: REF, refractory disease (*n* = 2); PR: partial response (*n* = 6); VGPR, very good partial response (*n* = 14); CR, complete response (*n* = 15). **(B)** MM patients with VGPR (*n* = 13) were grouped based on the absence (–IMiDs) (*n* = 4) or the presence (+IMiDs) (*n* = 9) of IMiDs in the induction chemotherapy. Responses significantly different by Mann-Whitney test are indicated as: ^*^p < 0.05 and ^**^0.001 < *p* < 0.01.

Lastly, to assess potential correlations between the immunological changes observed, the clinical status and the use of IMiDs in the induction chemotherapy, we analyzed the levels of cytokines in patients grouped according to their clinical status at 3 months after ASCT (Table 1). We found that IL-17, IL-22, TNF-α, IL-6, IL-23, and IL-1β were all significantly lower in patients with complete response compared to patients with refractory disease or partial response, whereas IFN-γ did not significantly change (Figure 4A). IL-6, IL-23, and IL-1β were also significantly lower in patients with complete response compared to those with very good partial response (Figure 4A).

As the cohort of patients that received IMiDs had better clinical outcome (complete response in 44 vs. 10% patients, respectively, see Table 1), to exclude that the immunological changes observed were simply the consequence of reduced tumor burden and not associated to IMiD-driven immune modulation, we compared the cytokine profile in IMiD-treated vs. untreated patients within patients with very good partial response (i.e., patients who had achieved very deep responses to therapy, yet still have some residual markers of disease). Although the number of patients compared is relatively small, we found that IL-17, IL-22, IL-6, and IL-1β were significantly lower in IMiD-treated compared to untreated patients (Figure 4B).

Together, these data suggest that the better clinical responses observed in IMiD-treated patients depend not only on the well-known anti-proliferative effects of IMiDs on myeloma cells (6) but possibly also on immune regulatory functions, among which the down-modulation of pro-tumor Th17-Th1, Th22 cells and their related cytokines.

## Conclusions

In this study we report that (i) pro-tumor Th17-Th1 and Th22 cells and their related cytokines in the BM after transplantation are lower in patients treated with IMiDs during induction chemotherapy compared to untreated patients and (ii) the levels of the same cytokines are lower in patients with favorable clinical outcome (i.e., complete response) compared to patients with persistent disease (i.e., refractory disease and partial response).

In agreement with previous reports (9, 13) we found higher numbers of IFN-γ secreting T cells in the BM of patients treated with IMiDs in the induction chemotherapy: however, even though the levels of IFN-γ in the BM sera were increased after transplantation compared with those at diagnosis, we did not find a significant correlation between the levels of IFN-γ and the clinical outcome after transplantation.

Conflicting results are reported concerning the role of IMiDs on Tregs: while *in vitro* lenalidomide reduced the number of Tregs (11), *in vivo* studies showed an increase in the number of Tregs in different clinical settings (13–15). In our study we did not find significant changes in the numbers of Tregs neither between diagnosis and after transplantation nor between IMiD-treated and untreated patients. Factors such as the clinical setting in different studies (i.e., consolidation and/or maintenance vs. induction chemotherapy) and the site of investigation (i.e., peripheral vs. BM blood) may account for these differences.

Our findings are particularly relevant because of the pro-tumor role of IL-17 and IL-22 in MM. IL-17 promotes MM cell growth/survival by interaction with the IL-17 receptor expressed on MM cells, and inhibits immune cell functions (28). In addition, expansion of Th17 cells correlates with development of bone lesions (29, 30), possibly through IL-17-induced upregulation in BM cells of RANKL, which is a differentiation factor for osteoclasts [rev. in (31)]. A reduction in the RANKL/osteoprotegerin ratio and bone osteolysis was reported in MM patients with relapsed/refractory disease treated with thalidomide-containing regimen (32, 33). In agreement with this report, we found that the percentage of nuclear magnetic resonance and/or positron emission tomography negative patients as from evaluation after transplantation was superior within the IMiD-treated compared to the untreated group (data not shown). Whether the effect on bone disease following treatment with IMiDs might at least partially depend on IL-17 modulation through the RANKL signaling as well as other mechanisms of bone remodeling [i.e., Wnt (34) and LIGHT/TNFSF14 (35) signaling pathways] might warrant further *ad hoc* investigation. Concerning IL-22, we previously reported that this cytokine directly increases MM cell growth and resistance to drug-induced cell death by binding to its IL22RA1 receptor, which is aberrantly expressed on a fraction of primary MM cells (26).

The immune regulation exerted by IMiDs has actually two sides: they co-stimulate activation of immune responses, as in hematologic cancers (6), but they can also dampen inflammatory reactions, as in autoimmune and inflammatory disorders (36). Such dual role conceivably depends on different immune cell types targeted, such as T and NK cells and antigen presenting cells, respectively (36). It is tempting to speculate that the use of IMiDs during induction chemotherapy might have favored the development and persistence of an anti-inflammatory BM milieu responsible for the reduced frequency of Th17-Th1 and Th22 cells, possibly through modulation of BM resident cells (i.e., mesenchymal stromal cells) and/or immune cells persisting in the BM or transferred within the autologous progenitor cell reinfusion.

In summary, our results contribute to the characterization of the immunomodulatory effects exerted by IMiDs in MM and should be taken into consideration for the implementation of new therapeutic strategies targeting IL-17 and IL-22 to be combined with drugs already used in MM treatment, especially in refractory/relapsed disease stages.

## Author Contributions

GDL designed research, performed research, analyzed data and wrote the manuscript. MM and FC contributed to patient selection and clinical data collection. SH performed research. CT contributed sample collection and storage. AMP contributed to statistical analysis design. CB contributed to data analysis. MPP designed research, analyzed data and wrote the manuscript.

### Conflict of Interest Statement

The authors declare that the research was conducted in the absence of any commercial or financial relationships that could be construed as a potential conflict of interest.

## Abbreviations

ASCT: autologous stem cell transplantation
BM: bone marrow
IMiDs: immunomodulatory drugs
MM: multiple myeloma
Treg: T regulatory cells.
