# Supplementary material for: Immunomodulatory Drugs in the Context of Autologous Hematopoietic Stem Cell Transplantation Associate With Reduced Pro-tumor T Cell Subsets in Multiple Myeloma
Source: Front Immunol. 2019 Jan 21;9:3171. doi: 10.3389/fimmu.2018.03171 (PMC6348257; doi:10.3389/fimmu.2018.03171)
Supplement: Supplementary file 1 [file Data_Sheet_1.docx]

***Supplementary Material***

**Immunomodulatory Drugs in the Context of Autologous Hematopoietic Stem Cell Transplantation Associate With Reduced Pro-tumor Th Cell subsets in Multiple Myeloma**

Giulia Di Lullo*, Magda Marcatti, Silvia Heltai, Cristina Tresoldi, Anna Maria Paganoni, Claudio Bordignon, Fabio Ciceri, Maria Pia Protti*

*Correspondence: Giulia Di Lullo, [dilullo.giulia@hsr.it](mailto:dilullo.giulia@hsr.it); Maria Pia Protti, [protti.mariapia@hsr.it](mailto:protti.mariapia@hsr.it)

**Supplementary Figures S1 and S2**


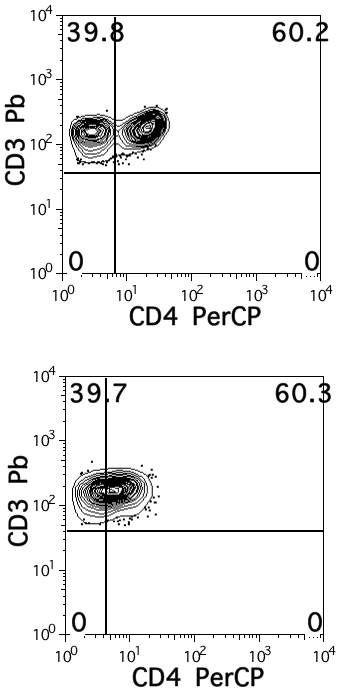

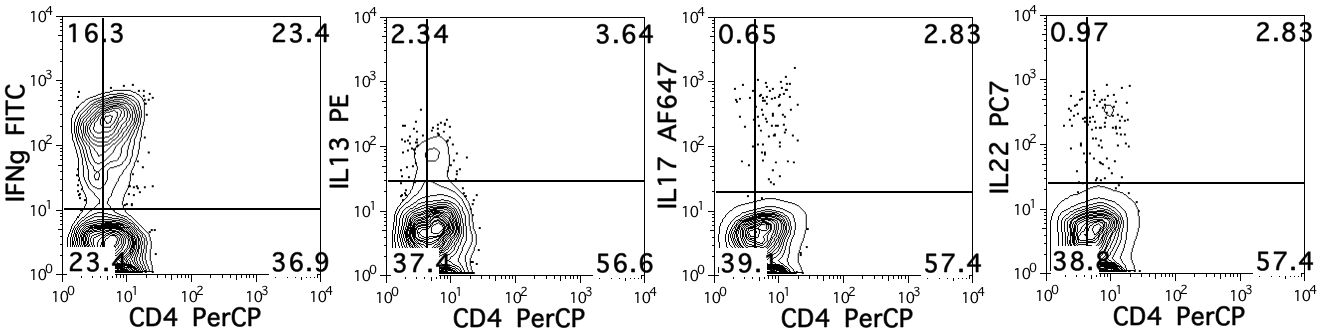

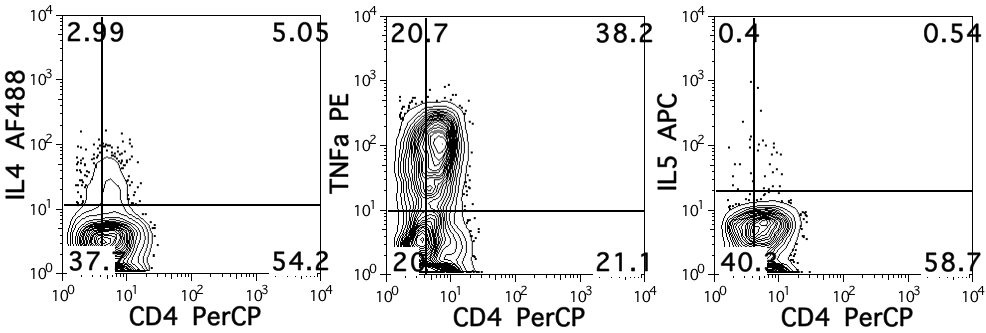


PMA + ionomycin

gated on CD3^+^ cells


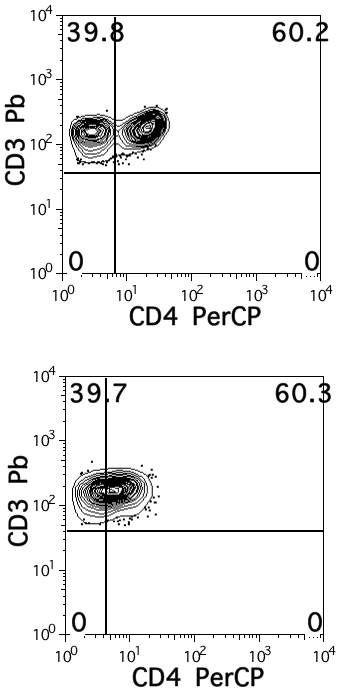


unstimulated

PMA + ionomycin (gated on CD3^+^ cells)

**SUPPLEMENTARY FIGURE S1. (A)** Representative dot plots of CD3 and CD4 expression on unstimulated and PMA + ionomycin-stimulated BM mononuclear cells from one patient of the study. Cells were treated or not with PMA + ionomycin for 5 hours and intracellular cytokine staining was performed as detailed in Materials and Methods. (**B**) Representative expression of CD4 and the indicated cytokines in PMA + ionomycin-stimulated BM mononuclear cells from the same patient. Plots in (**A**) and (**B**) are gated on CD3^+^ lymphocytes.

**A**

**B**

**SUPPLEMENTARY FIGURE S2.** Cytokines measured in paired samples of BM sera collected at diagnosis and at 3 months after ASCT. (A) IFN-γ, IL-13, IL-4 and IL-5 (n=24). TNF-α, IL-17, and IL-22 (n=25). (B) IL-6, IL-23 and IL-1β (n=25). Responses significantly different by Wilcoxon Signed-Rank Test are indicated as: *p<0.05, **0.001<p<0.01 and ***p<0.001. ns: not significant.

**A**

**B**
